# Calibration of the food parenting practice (FPP) item bank: tools for improving the measurement of food parenting practices of parents of 5–12-year-old children

**DOI:** 10.1186/s12966-020-01049-9

**Published:** 2020-11-16

**Authors:** Louise C. Mâsse, Teresia M. O’Connor, Yingyi Lin, Sheryl O. Hughes, Claire N. Tugault-Lafleur, Tom Baranowski, Mark R. Beauchamp

**Affiliations:** 1 BC Children’s Hospital Research Institute, School of Population and Public Health University of British Columbia, F508-4490 Oak Street, Vancouver, BC V6H 3V4 Canada; 2 USDA/ARS Children’s Nutrition Research Center, Baylor College of Medicine, 1100 Bates St, Houston, TX 77030 USA; 3 School of Kinesiology, University of British Columbia, 210-6081 University Blvd, Vancouver, BC V6T 1Z1 Canada

**Keywords:** Food parenting practices, Parents, Children, Psychometric, Validity, Measurement, Questionnaire

## Abstract

**Purpose:**

There has been a call to improve measurement rigour and standardization of food parenting practices measures, as well as aligning the measurement of food parenting practices with the parenting literature. Drawing from an expert-informed conceptual framework assessing three key domains of food parenting practices (autonomy promotion, control, and structure), this study combined factor analytic methods with Item Response Modeling (IRM) methodology to psychometrically validate responses to the Food Parenting Practice item bank.

**Methods:**

A sample of 799 Canadian parents of 5–12-year-old children completed the Food Parenting Practice item bank (129 items measuring 17 constructs). The factorial structure of the responses to the item bank was assessed with confirmatory factor analysis (CFA), confirmatory bi-factor item analysis, and IRM. Following these analyses, differential Item Functioning (DIF) and Differential Response Functioning (DRF) analyses were then used to test invariance properties by parents’ sex, income and ethnicity. Finally, the efficiency of the item bank was examined using computerized adaptive testing simulations to identify the items to include in a short form.

**Results:**

Overall, the expert-informed conceptual framework was predominantly supported by the CFA as it retained the same 17 constructs included in the conceptual framework with the exception of the access/availability and permissive constructs which were respectively renamed covert control and accommodating the child to better reflect the content of the final solution. The bi-factor item analyses and IRM analyses revealed that the solution could be simplified to 11 unidimensional constructs and the full item bank included 86-items (empirical reliability from 0.78 to 0.96, except for 1 construct) and the short form had 48 items.

**Conclusion:**

Overall the food parenting practice item bank has excellent psychometric properties. The item bank includes an expanded version and short version to meet various study needs. This study provides more efficient tools for assessing how food parenting practices influence child dietary behaviours. Next steps are to use the IRM calibrated item bank and draw on computerized adaptive testing methodology to administer the item bank and provide flexibility in item selection.

**Supplementary Information:**

The online version contains supplementary material available at 10.1186/s12966-020-01049-9.

## Introduction

Children’s eating behaviours and dietary intakes are influenced by numerous interacting factors at the individual, household, and broader community and societal levels [1, 2]. However, the home and family environment are thought to play a particularly important role in influencing children’s dietary patterns and weight status. Parents act as gatekeepers and strongly influence the home’s physical and social environment through their parenting and dietary behaviours [3, 4].

Parenting practices refer to the specific goal directed strategies that parents use to influence their child’s behaviours, such as eating [5]. Food parenting practices represent a wide array of techniques and behaviours used by parents to influence children’s diet and/or weight [6, 7]. Several salient constructs have been identified including pressure to eat, restriction, monitoring child intake, use of food as a reward, role modelling, food preparation practices, and involving the child in food planning and preparation [6]. Reviews on the determinants of children’s fruit and vegetable consumption have shown that some parenting practice constructs such as parental modelling, availability of vegetables and fruit, food rules and encouragement are consistently associated with higher fruit and vegetable intake [8–12]. However, some of these reviews [8, 10] also point to a lack of standardization in how each construct is measured across studies.

A growing number of instruments are available to evaluate how parents influence their children’s eating behaviours. A 2013 review paper identified 57 unique instruments to measure food parenting practices [13]. Three years later, another review identified over 75 published articles concerned with the development of unique food parenting instruments [14]. There have been several calls to better align instruments evaluating food parenting practices with the parenting literature as a way to bring more rigour and standardization to the measurement of food parenting constructs [7, 15, 16]. In response to these calls, our team engaged with 25 experts from eight countries and drew on concept mapping methods to develop a food parenting practice conceptual framework for 5–12 year old children [17]. The experts were asked to sort 110 food parenting practice concepts that were derived from the literature review and a semi-qualitative study of US and Canadian parents, into meaningful groups or constructs [17]. A non-parametric multidimensional scaling analyses with feedback from the expert panel identified a comprehensive concept map that contained 17 constructs based on three key domains of food parenting practices: autonomy promotion, control, and structure. Building on this conceptual framework [17], the purpose of this study was to assess the psychometric properties of responses to the food parenting practices item bank. Specifically, this study aimed to: 1) validate the factor structure of responses derived from the food parenting practices item bank using the published food parenting practices conceptual framework [17]; 2) examine whether the psychometric properties of the constructs assessed by the food parenting practices item bank are similar by parents’ sex, income, and ethnicity; and 3) determine the efficiency of the item bank and whether the constructs can be assessed with fewer items. The ultimate objective of this work is to develop a repository of calibrated items to standardize measurements of food parenting practices for 5–12-year-old children – a period that coincides with children’s entry into the school system (pre-school to elementary school).

In this paper, we draw on procedures developed by the NIH PROMIS initiative, a measurement initiative that set the methodology for assessing the reliability and validity of responses derived from an item bank [18, 19]. Specifically, following the NIH PROMIS methods this study used both classical (Confirmatory Factor Analysis - CFA) and advanced psychometric (confirmatory bi-factor item analyses with Item Response Modeling - IRM) methods to develop a more parsimonious way of measuring food parenting practices of parents of 5–12 year-old children – a period in which children remain highly influenced by their caregivers. As the current conceptual framework of food parenting practices [17] includes 17 constructs, it is unlikely that many studies will be able to assess all these constructs. Therefore, there is a need to determine whether our conceptual framework can be simplified. While CFA provides an initial assessment of the psychometric properties of a measure, CFA does not provide the process for testing whether strongly related constructs within each domain of food parenting practices are strongly related to a common general construct. Hence, this study used both confirmatory bi-factor item analyses and IRM to test the psychometric properties of the food parenting practice item bank to identify a simpler structure and develop shorter measures.

## Methods

### Participants

Parents of 5–12-year-old children were recruited by a Canadian marketing research company (Insight West, British Columbia, Canada) using their web-based panel members. Potential participants were first screened by Insight West to determine their eligibility for this study which included: being a parent or guardian of a 5–12-year-old child and not having a child with a condition that would severely limit their diet. A quota sampling approach was used to ensure adequate representation by parents’ sex, income (using the 2015 median income of Canadian parents), education, and ethnicity (Caucasian, East/Southeast Asian, South/West Asian and others) as well as by characteristics of the child (age group - 5 to 9 and 10 to 12 years of age). The sampling strategy aimed for a 50% split by parents’ sex and ethnicity (i.e., 50% non-Caucasian) and child age group. For parent’s education (bachelor and above or below) and income (CAD $80,000 and above or below, where $80,000 represent the median income of dual household families) the quota was relaxed to allow for a 40 to 60% split. A quota sampling strategy was used to ensure enough sample diversity was present to test whether the psychometric properties of the food parenting item bank are stable in relation to characteristics of the respondents. Note that only one member of a household participated in the study and as such, the mothers and fathers that participated were not from the same household.

In total, 799 parents met the eligibility criteria and completed the online questionnaire (see Table 1). Data were collected from September 2017 to January 2018 and the protocol was approved by the University of British Columbia Research Ethics Board. Potential participants consented to be part of the web-based panel and consented to be part of the study before they completed the online questionnaire.

**Table 1:** Demographic characteristics of participants (*N* = 799)

		% or Mean (SD)
Parent Sex (N = 799) Female		50.1%
Parent Age (N = 799)		33.1 (8.5)
Marital status (N = 799)	Married or common law	86.0%
	Separated or divorced or widowed	9.0%
	Never married	5.0%
Ethnicity (N = 799)	Caucasian	50.7%
	Asian	22.3%
	South Asian	15.6%
	Other	11.4%
Education (*N* = 600)	High school or less	13.0 .0%
	Certificate non-university or some college or university	30.2%
	Bachelor’s degree	30.8%
	Postgraduate degree	19.0%
	Professional degree	6.0%
Income (N = 799)	Less than $50,000 Cdn	21.9%
	$50,000–69,999 Cdn	20.7%
	$70,000–79,999 Cdn	11.4%
	$80,000–99,999 Cdn	18.7%
	$100,000–124,999 Cdn	12.3%
	$125,000 Cdn or higher	15.2%
Child Sex (N = 799) Female		44.1%
Child age (N = 799)	5–9 year old	49.8%
	10–12 year old	50.2%

### Measures

Development of the item bank followed the process used by the NIH-PROMIS initiative [19]. which consists of: 1) identifying existing items from the literature and supplementing the item pool, 2) following a binning (i.e., grouping items that measure the same construct and area) and winnowing (i.e., selecting the items that best represent the constrict and area) process to reduce the pool of items into representative items as well as assigning it to the proper dimension it assesses; 3) standardizing the items; 4) involving experts in the review process; 5) conducting cognitive interviews with participants, and 6) finalizing the item bank [17].

As part of the development of the food parenting practices conceptual framework, our team conducted a literature review of food parenting practices measures and retrieved 1392 items from 79 measures [14]. In addition, we conducted an online open-ended qualitative survey among 135 parents which resulted in identifying 1985 unique food parenting practices [14]. Using the NIH-PROMIS binning and winnowing process, the items gathered from the literature and parent responses were reduced into unique representative items which were then reviewed by 25 experts using concept mapping analyses to derive the conceptual framework for the food parenting practices item bank (see [17] for further details). Using the expert-endorsed conceptual framework, the items from the literature and the parent responses were used as input to develop the food parenting practices item bank. LCM and TO operationalized the content of the item bank to ensure the constructs matched the operational definition of the conceptual framework and selected the most appropriate items. Items were selected to represent the breath of the content but eliminated redundancy in content. As the items were taken from various sources, the items and responses format were standardized. The initial item bank was reviewed by the larger team of investigators (MB, SH, and TB) and were iteratively modified until the team converged and agreed on the content of the item bank.

The resulting item bank included 129 items covering the 17 constructs that measured three key domains of parenting practices, namely autonomy promotion, control, and structure. The autonomy support domain included three constructs (22 items) that measured: 1) *Child involvement* – 4 items measuring whether parents actively involve their child in meal preparation; 2) *Encourage healthy eating* – 5 items measuring whether parents suggest their child eat healthy foods without being forceful; and 3) *Education/reasoning* – 13 items measuring whether parents educate or use games to teach their child about the nutritional value of foods and beverages.

The control domain included five constructs (37 items) that measured: 1) *Restriction for weight* – 5 items measuring whether certain foods or beverages are restricted or discouraged to control their child’s weight; 2) *Using food to control negative emotion* – 5 items measuring whether foods are used to manage their child’s negative emotions (e.g., providing food for comfort when the child is upset or hurt); 3) *Threats and bribes* – 10 items measuring whether food is taken away as a results of bad behaviour or used as a reward for good behaviour; 4) *Pressure to eat* – 12 items measuring whether their child is forced or told to eat certain foods and/or clean their plate without taking into account the satiety level of the child; and finally, 5) *Intrusive control* – 5 items measuring whether parents dictate what their child should eat and get their child to eat things without their knowledge.

The structure domain included nine constructs (70 items) that measured: 1) *Modeling* – 5 items measuring whether parents engaged in specific food-related behaviours that may be emulated by their child; 2) *Prompt to eat* – 6 items measuring whether parents suggest but do not force their child to eat certain foods; 3) *Food preparation* – 10 items measuring the strategies parents use to plan and make healthful meals or snacks; 4) *Exposure to variety/selection* – 6 items measuring whether parents repeatedly exposes their child to nutritious and varied foods; 5) *Rules and limits* – 12 items measuring whether parents have expectations or boundaries around the quantity or quality of foods consumed; 6) *Redirection and negotiation* – 5 items measuring whether parents engage with their child to distract or negotiate to take their child’s mind off consuming certain foods or beverages without being forceful; 7) *Meal routines* – 9 items measuring whether parents have set routines around meal times and snacks; 8) *Food Accessibility/availability* – 9 items measuring whether certain foods are brought in the home and whether it is readily accessible; and 9) *Permissive feeding* – 8 items measuring whether parents allow their child to control the quantity and quality of foods eaten without setting expectations.

The food parenting practices item bank was then cognitively tested with 11 parents using a combination of think-aloud and probing protocols [20]. The think-aloud protocol asked parents to read each item, paraphrase the question, and explain why they chose a specific response. The probing protocol asked parents to complete one section of questions first. Then parents were asked probing questions to test their understanding of the item(s) they struggled to answer. The items were iteratively modified during this process and reviewed by the research team before administration.

As the food parenting practices questionnaire was large (129 items), the questionnaire was administered to the same sample of parents in two waves approximately one week apart (i.e., receiving half of the items on the first week and the remaining week receiving the other items). Items were administered online in blocks of about 10–20 items which were randomly administered to change the order of administration.

### Analyses

**Fig. 1:**
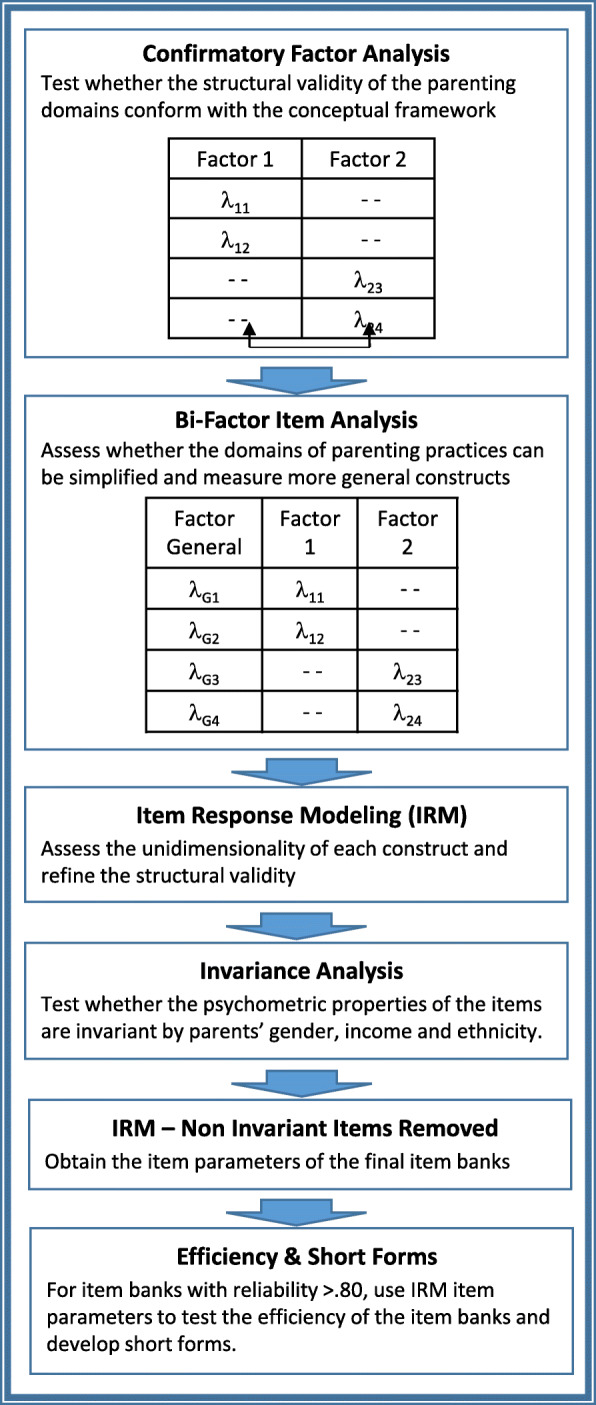
Analytical steps

Figure 1 summarizes the analytical steps used to assess the psychometric properties of the food parenting practices item bank.

#### Validating the factorial structure of the food parenting practices item bank (aim 1)

This was a three-step process which started with a CFA, followed by a confirmatory bi-factor item analysis, and then an IRM analysis. As the CFAs were followed by bi-factor item analyses, the analyses had to be conducted for each of the three domains of food parenting practices separately (autonomy promotion, control, and structure). The CFA assessed whether the factorial structure of measures derived from the food parenting practices item bank conformed with the initial Food Parenting Practices conceptual framework which was described in the measurement section. If the constructs within a domain were highly correlated (r > .70), the CFA was followed by a confirmatory bi-factor item analysis to determine whether a simpler structure exists. These analyses tested whether a general construct could be used to simplify the structure of the initial conceptual framework.

The CFA and confirmatory bi-factor item analyses used the MPlus version 8 software [21]. Given the ordinal nature of the response, the weighted least squares mean and variance-adjusted (WLSMV) estimation method was used with a full information procedure [22]. To assess CFA model fit the following indices were evaluated: Steiger’s Root Mean Square Error of Approximation (RMSEA), with an upper value of 0.08 to 0.10 indicative of a good fit; the Comparative Fit Index (CFI) with values ≥0.95 suggestive of an acceptable fit; and the weighted root mean square residual (WRMR) with a value closer to 1 suggestive of an excellent fit [23–26]. For the confirmatory bi-factor item analyses, the Explained Common Variance for a Single Item (I-ECV) index was used to assess whether the items loaded on the general construct [I-ECV = (loadings of the general construct)^2^ / ((loadings of the general construct)^2^ + (loadings of the specific construct)^2^)] [27]. Item with I-ECV ≥0.70 and/or factor loading on the general construct ≥.50 were candidate items for the general construct. In rare instances, an item with lower I-ECV was kept for conceptual reasons (i.e., thought to represent an important aspect of the construct not measured by other items).

As a final step in the assessment of the psychometric property of the food parenting practices item bank, the constructs were analyzed with Item Response Modeling (IRM) using the MIRT package in R software [28]. These analyses served to confirm the unidimensionality of the initial or simplified factor structure, to confirm whether any items remaining in the item bank were locally dependent. Local dependence occurs when the items are similar and found to not contribute broader content to the construct. Overall fit of the IRM model is assessed with the M^2^ statistic (which is a variant of the χ^2^ statistic), RMSEA, CFI, and Standardized Root Mean Square residuals (SRMR – where values between 0.05 to 0.08 are indicative of a good fit) [29]. In the context of this study, evaluation of the fit statistics of the IRM analyses was secondary, as overall fit was assessed with either CFA and/or confirmatory bi-factor item analyses. Instead, the primary aims of these analyses were to assess local dependence after the IRM model was fitted (which is considered meaningful when any residual correlations are greater than .25). Any items found to be locally dependent were evaluated to determine whether its content conceptually overlapped with another item. If it did, one item in a pair was deleted and the model was re-run to determine whether any local dependence remained. Cronbach alphas and IRM reliability (similar to Cronbach alpha except it assumes the data are ordinal) of the final scales were reported.

#### Invariance analyses (aim 2)

Once the factor structure of the food parenting practices item bank was validated, the next step was to assess whether the psychometric properties of the items were invariant by parents’ sex, income, and ethnicity. Following Chalmers’s procedures [28], the analyses assessed whether any items had any significant Differential Item Functioning (DIF). Since these analyses are highly affected by sample size and the DIF identified may not be meaningful, the analyses were followed by a Differential Response Functioning (DRF) analyses [28]. This analysis tested for two types of DRF, whether the DRF was constant or inconsistent across the scores on a given construct. The DIF and DRF analyses examined the invariance properties of the items by parents’ sex (male vs female), income (low vs high based on the 2015 median income of Canadian parents), and ethnicity (Caucasian vs non-Caucasian). Any items that exhibited significant DRF (*p* < .01) were removed from the item bank and IRM results were re-run without these items.

#### Efficiency and short forms (aim 3)

The last step in the analyses was to determine the efficiency of the item bank that had reliability > 0.80. The IRM results, specifically the item parameters (i.e., item discrimination and difficulty parameters) served as input for estimating the efficiency and the number of items needed to retain a reliability of 0.80. The output from the IRM analyses were imported into the Firestar software [30]. Firestar uses Computerized Adaptive Testing simulations for graded response models to test the efficiency of the item bank and employs the maximum posterior weighted information function to determine the items that maximize the reliability of the item bank.

## Results

### Autonomy promotion domain of food parenting practices

The hypothesized 3-factor model for the Autonomy Promotion domain (child involvement, encourage healthy eating, and education/reasoning) did not fit the data (Table 2). However, an adequate fit was achieved with a revised 3-factor model that deleted four highly correlated items. Two of the constructs within the Autonomy Promotion domain were highly correlated (r = 0.88 between encourage healthy eating and education/reasoning). Therefore, a bi-factor item analysis was run to determine whether these two constructs measured a general construct. The bi-factor analysis supported combining the encourage and education/reasoning constructs (14 items) and keeping the child involvement construct (4 items) as a separate factor. As this made conceptual sense, the combined general construct was labeled autonomy support and included 18 items (see solution in Table 3 and Additional file 1: Appendix A). The IRM analyses supported the factorial structure of the constructs and no DRF were found.).

**Table 2:** Fit statistics for the Confirmatory Factor Analyses (CFA), Bi-factor Item Analyses (Bi-Factor), Item, and Response Modeling Analyses (IRM) for the three domains of food parenting practices

**AUTONOMY PROMOTION FOOD PARENTING DOMAIN**					
**CFA / Bi-Factor**	**χ**^**2**^	**RMSEA [90% CI]**	**CFI**	**WRMR**	
CFA – Hypothesized 3-factor model	x^2^(df = 206) = 2294, *p* < .05	.113 [.111–.117]	.88	2.59	Poor fit
CFA – Revised 3-factor model	x^2^(df = 132) = 782, p < .05	.079 [.073–.084]	.95	1.68	Adequate fit
Bi-Factor: Autonomy support	x^2^(df = 117) = 658 p < .05	.076[.071–.082]	.96	1.32	Adequate fit
**IRM**	**M**^**2**^	**RMSEA [90% CI]**	**CFI**	**SRMR**	**LD / DRF**
IRM: Autonomy support	M^2^(df = 35) = 118 p < .05	.063 [.051–.076]	.92	.07	Borderline LD No DRF
IRM: Child involvement	Fit indices not computed when items are less than 10				No LD / No DRF
**CONTROL FOOD PARENTING DOMAIN**					
**CFA / Bi-Factor**	**χ**^**2**^	**RMSEA [90% CI]**	**CFI**	**WRMR**	
CFA – Hypothesized 5-Factor Model	x^2^(df = 619) = 5893, p < .05	.103 [.101–.106]	.89	2.88	Poor fit
CFA – Revised 5-Factor Model	x^2^(df = 424) = 2082, p < .05	.070 [.067–.073]	.96	1.70	Adequate fit
Bi-Factor: Coercive control	x^2^(df = 403) = 2514, p < .05	.081 [.078–.084];	.95	1.89	Adequate fit
**IRM**	**M**^**2**^	**RMSEA [90% CI]**	**CFI**	**SRMR**	**LD / DRF**
IRM: Coercive control	M^2^(df = 161) = 910, p < .05	.076 [.071–.081]	.96	.05	No LD /No DRF
IRM: Restrict for weight	Fit indices not computed when items are less than 10				1 LD / No DRF
**STRUCTURE FOOD PARENTING DOMAIN**					
**CFA / Bi-Factor**	**χ**^**2**^	**RMSEA [90% CI]**	**CFI**	**WRMR**	
CFA – Hypothesized 9-factor model	x^2^(df = 2309) = 14,889, p < .05	.083 ([.081–.084]	.61	3.30	Poor fit
CFA – Revised 9-factor model	x^2^(df = 743) = 2428, p < .05	.053 [.051–.056]	.91	1.67	Adequate fit
Bi-Factor: Nondirective support	x^2^(df = 12) = 56, p < .05	.079 [.059–.100]	.99	0.61	Adequate fit
Bi-Factor: Provide healthy eating opportunities	x^2^(df = 18) = 47, p < .05	.045 [.030–.061]	.99	0.56	Adequate fit
**IRM**	**M**^**2**^	**RMSEA [90% CI]**	**CFI**	**SRMR**	**LD / DRF**
IRM Nondirective support	Fit indices not computed when items are less than 10				Borderline LD No DRF
IRM Provide healthy eating opportunities	Fit indices not computed when items are less than 10				No LD 1 DRF by ethnicity
IRM Rules and limits	Fit indices not computed when items are less than 10				No LD / No DRF
IRM Redirection	Too few items for IRM and DRF analyses				
IRM Meal routines	Fit indices not computed when items are less than 10				No LD / No DRF
IRM Covert control	Fit indices not computed when items are less than 10				1 LD / No DRF
IRM Accommodating the child	Fit indices not computed when items are less than 10				No LD / No DRF

χ^2^ / M^2^ = Chi-square; RMSEA = Root Mean Square Error of Approximation; 90% CI: 90% Confidence Interval, where upper 90%CI less than .10 is indicative of a good fit; CFI = Comparative Fit Index, where values between .90 to 95 are indicated of a good fit; WRMR = Weighted Room Mean Residual, where values less than 2.0 are indicate of a good fit. SRMR = Standardized Root Mean Residual, where values less than .08 are indicative of a good fit; LD = Local Dependence; DRF: Differential Response Functioning

**Table 3:** Results from the Confirmatory Factor Analyses (CFA), Bi-factor item analyses, and Item Response Modeling analyses ^a^

Items	CFA		Bi-Factor Item Analysis			Drop code
	Constructs (Cronbach α)	λ	Constructs	λ	I-ECV	
**AUTONOMY PROMOTION FOOD PARENTING DOMAIN**						
1 Have your child help prepare dinner meals	Child Involvement (.75)	.87	Child involvement	.22	.06	
2 Have your child help you prepare vegetable dishes		.93		.27	.09	
3 Give your child a choice of veggies to eat at dinner		.59		.27	.24	
4 Ask your child’s opinion about what to make for meals		.48		.26	.37	
5 Serve healthy foods (veggies) in the way child likes	Encourage (.77)	.65	Autonomy support	.59	.83	
8 Tell child you like it and that he or she might like it also		.83		.74	.78	
9 Say something nice if child taste a NEW vegetable / food		.82		.72	.78	
10 Tell your child that colorful veggies are healthier	Education / reasoning (.91)	.72		.69	.91	
11 Read food labels to help child choose healthier options		.67		.63	.81	
12 Explain that eating healthy food gives more energy		.80		.85	.96	
13 Explain how good it is to eat or taste vegetable		.83		.87	.97	
14 Will do better in school by eating healthier/veggies		.75		.74	.98	
16 Teach your child to stop eating when full		.66		.59	.65	
17 Give child ideas on how to make healthy food choices		.79		.76	.88	
18 Explain your reasons for food rules tell		.76		.73	.89	
20 Explain that treats are sometimes food		.63		.62	.97	
21 Teach your child to eat food from all the food groups		.65		.63	.91	
22 Help child set goals to eat more veggies/healthier		.76		.70	.75	
**CONTROL FOOD PARENTING DOMAIN**						
1 Keep away from specific sweet or salty treats	Restriction for weight (.80)	.52	Restriction for weight	.34	.40	
2 Keep a record of how much your child eats		.86		.62	.55	
3 Not allow child to take second helpings at dinner		.84		.60	.52	
4 Decide how much/how often your child eats		.53		.34	.26	LD
5 Talk to your child about dieting/loosing weight		.88		.63	.55	
6 Give sweet or salty treat to make your child feel	Use food to control negative emotions (.95)	.90	Coercive control	.81	.79	
7 Offer a treat when child is worried/stressed		.92		.82	.77	
8 Offer a treat to calm your child down		.95		.86	.84	
9 Give treat when talking/doing chores		.94		.86	.86	
10 Give treat to keep when child is bored		.92		.84	.82	
11 Child gets dessert if tastes veggies served	Threats & bribes (.94)	.75		.67	.69	
12 Promise child dessert if finish meal		.76		.68	.70	
13 Send child to room if does not finish meal		.90		.89	.98	
14 Reduce TV/ videogame if child does not finish meal		.80		.74	.81	
15 Reward good behaviours with a sweet or salty treat		.76		.71	.84	
16 Take away dessert for bad behaviour		.79		.74	.84	
17 Offer a treat to make child do something		.88		.85	.96	
18 Take away TV/videogame if not eat veggies		.90		.86	.90	
19 Send child to room for refusing to eat veggies		.93		.91	.99	
20 Tell child will be punished for eating without asking		.81		.80	.99	
25 Make child stay at table until all the food is eaten	Pressure to eat (.79)	.49		.39	.96	
26 Make child eat more even if s/he says “I am full”		.72		.51	.42^b^	
27 Physically struggle with child to eat meal		.88		.65	.54	I-ECV
28 Guilt child into eating his or her meal		.84		.62	.50	I-ECV
29 Show disappointment if child does not eat veggies		.73		.53	.48	I-ECV
30 Make sure child eats all veggies at dinner time		.64		.52	.99	
32 You force child to eat some veggies every day		.45		.37	.97	
34 Hide veggies in the food you serve	Intrusive control (.79)	.68		.64	.96	
35 Make child feel bad about what s/he eats		.84		.78	.92	
36 Not allow child to have a treat at parties		.78		.72	.82	
37 Make a lighter meal, if child ate more earlier		.66		.61	.74	
**STRUCTURE FOOD PARENTING DOMAIN**						
1 Eat healthy snacks when child is around	Modeling (.69)	.66	Non-directive support	.57	.87	
3 Eat healthy portions while with child		.68		.47	.18^b^	
5 Enjoy eating veggies when with child		.76		.64	.86	
7 Encourage child to eat food as served	Prompt to eat (.83)	.68		.70	1.00	
8 Encourage child to eat more if not full		.73		.68	.80	
9 Get child to eat more veggies		.79		.77	.97	
10 Encourage child to eat more		.76		.72	.75	
11 Get child to taste a new veggies		.74		.78	.95	
12 Family meals prepared from scratch	Meal preparation (.68)	.59	Providing healthy eating opportunities	.59	.94	
14 Serve veggies your child likes		.72		.68	.95	
15 Serve colourful veggies with meals		.77		.81	.90	
22 Serve a vegetable multiple times	Exposure to variety / selection (.75)	.67		.59	.96	
23 Serve child at least 2 different veggies		.68		.71	.99	
24 Serve child at least 5 different veggies		.77		.77	.75	
25 Serve at least 5 different fruits		.70		.64	.77	
26 Consistently served veggies since young		.45		.47	1.00	
27 Foods from different countries / cultures		.47		.42	.89	DRF
28 Know how many treats child has	Rules and limits (.86)	.69	Rules and limits	NA	NA	
29 Limit sweet or salty treats		.81		NA	NA	
31 Do not let child drink soda/sugary drinks		.61		NA	NA	
33 Limit size of treats child eats		.82		NA	NA	
34 Expect child to eat what you serve		.40		NA	NA	
35 If child eats a treat, expect next healthy		.72		NA	NA	
36 Expect child to drink mostly water/milk		.68		NA	NA	
37 Ask those who care for child to limit treats		.64		NA	NA	
39 Child ask permission before eating treat		.73		NA	NA	
41 Encourage to take small portion for treats	Redirection (.61)	.72	Redirection	NA	NA	
44 Talk/agree about treat options with child		.66		NA	NA	
47 Make child eat dinner meals at the table	Meal routines (.74)	.81	Meal routines	NA	NA	
50 Eat dinner together as a family		.73		NA	NA	
52 No play/talk/text while eating		.70		NA	NA	
53 No TV while eating		.66		NA	NA	
58 Keep treats out of your child’s reach	Covert control (.76)	.80	Covert control	NA	NA	
59 Hide sugary drinks so child cannot find		.81		NA	NA	
61 Throw away leftover treats		.68		NA	NA	
62 Not bring sugary drinks into home		.59		NA	NA	LD^b^
63 Eat out or get take-out food for meals	Accommodating the child (.81)	.56	Accommodating the child	NA	NA	
65 Give in and let child have dessert		.74		NA	NA	
67 Allow child to skip meals		.76		NA	NA	
69 Make only the foods child asks for meals		.73		NA	NA	
70 Buy a treat to fill child when on the go		.76		NA	NA	

^a^An expanded version of this table (showing correlations among constructs and content drop) is shown in Additional file 1: Appendix A

Drop code: DRF: Deleted since item is not invariant (significant Differential Response Functioning); I-ECV (explained common variance for a single item) is less than .50 I-ECV; and LD = LD Local dependence

^b^Kept from a content perspective even though the I-ECV is low or LD is present

### Control domain of food parenting practices

The hypothesized 5-factor model for the Control domain (restriction for weight, using food to control negative emotions, threats and bribes, pressure to eat, and intrusive control) did not fit the data (Table 2). A revised model that retained 5 factors but dropped 6 items (as the items had high correlated error terms with other items or did not load highly on the hypothesized factors) resulted in an adequate fit (Table 2). As the five constructs were highly correlated (correlations ranging from .55 to .89), a bi-factor item analysis was run forcing all the items to load on one general construct. The bi-factor item analysis solution had an adequate fit but only four of the five constructs loaded onto the combined general construct labelled *coercive control* (namely pressure to eat, using food to control for negative emotions, threats and bribes, and intrusive control). The restriction for weight remained its own construct. The IRM analyses supported the unidimensionality of the directive control (23 items) construct and dropped one item from the restriction for weight (4 items) construct. No items were found to have any DRF (see Table 2). Solution is shown in Table 3 and Additional file 1: Appendix C.

### Structure domain of food parenting practices

As shown in Table 2, the hypothesized 9-factor model for the Structure domain (prompt to eat, rules and limits, permissive feeding, food accessibility/availability, food preparation, modeling, exposure to variety/selection, meal routines and redirection and negotiation) did not fit the data. Any attempts to modify the hypothesized model by evaluating the modification indices did not yield an adequate fit; therefore, each construct was evaluated separately and then a larger model was rerun. A revised 9-factor model that dropped 29 items had an adequate fit (Table 2). The revised solution retained the factorial structure of the hypothesized solution, except the food accessibility/availability and the permissive constructs were relabeled as *covert control* and *accommodate the child* respectively to better reflect the items.

Since the correlations among some constructs were high (r > 0.70) for the CFA solutions, two bi-factor item analyses were run to verify whether a simpler solution existed. The first bi-factor item analysis that combined modeling and prompt to eat (r = 0.79) was supported (see Table 2) and the combined general construct was labeled as *nondirective support*. The second bi-factor analysis that combined meal preparation and exposure to variety/selection (r = 0.97) was supported (see Table 2) and the general construct was labeled *Providing healthy eating opportunities*.

As a result of the IRM and invariance analyses, one more item was dropped. Item 27, which assessed whether the child is exposed to foods from different countries or cultures, was dropped because DRF was significant by ethnicity. The solution is shown in Table 3 and Additional file 1: Appendix A.

For comparison purposes, Table 4 shows how the food parenting practice item bank results align with our conceptual framework based on expert input [17] and Vaughn and colleagues content map [6]. Overall, the CFA results show the findings align closely with our conceptual framework with the exception of two constructs that were renamed to better reflect the content of these constructs (namely covert control and accommodate the child). Importantly, Table 4 shows how the structure of our conceptual framework could be further simplified by measuring broader constructs identified by the bi-factor item analyses and IRM analyses in 11 constructs (see Table 4).

**Table 4:** An overview of how the psychometric findings align with our Food Parenting Practices conceptual framework and Vaughn and colleagues Content Map

Vaughn and Colleagues Content Map [6]		O’Connor and colleagues Our conceptual Framework [17]		Food Parenting Practices Item Bank Psychometric Analyses	
Domain	Constructs	Domain	Constructs	Confirmatory Factor Analyses Constructs	Bi-Factor Item Analyses and Item Response Modeling Constructs
**Autonomy support or promotion**	Child involvement	**Autonomy promotion**	Child involvement	Child involvement	Child involvement
	Encouragement		Encourage healthy eating	Encourage	Autonomy support
	Praise				
	Nutrition education		Education / Reasoning	Education / reasoning	
	Reasoning				
	Negotiation				
**Coercive control**	Restriction	**Control**	Restriction for weight	Restriction for weight	Restriction for weight
	Using food to control negative emotions		Using food to control negative emotions	Using food to control negative emotions	Coercive control
	Threats and bribes		Threats and bribes	Threats and bribes	
	Pressure to eat		Pressure to eat	Pressure to eat	
			Intrusive control	Intrusive control	
**Structure**	Modeling	**Structure**	Modeling	Modeling	Nondirective support
			Prompt to eat	Prompt to eat	
	Food preparation		Food preparation	Meal preparation	Provide healthy eating opportunities
			Exposure to variety / selection	Exposure to variety / selection	
	Monitoring		Rules and limits	Rules and limits	Rules and limits
	Rules and limits				
	Limited / guided choice		Redirection and negotiation	Redirection	Redirection
	Meals and snack routines		Meal routines	Meal routines	Meal routines
	Food accessibility		Food accessibility / availability	Covert control	Covert control
	Food availability				
	Unstructured practices		Permissive feeding	Accommodate the child	Accommodate the child

### Final item bank and efficiency analysis

Table 5 presents the results of the efficiency analyses. In total, five constructs could be efficiently measured with fewer items and retained an IRM reliability of 0.80: autonomy support, coercive control, nondirective support, providing healthy eating opportunities, and rules and limits. Table 5 presents a list of items included in the full food parenting practices item bank and the items included in the short form based on the results of the efficiency analyses (see Additional file 1: Appendix B for a copy of the instrument).

**Table 5:** Items included in the Item bank by domains of food parenting practices and constructs showing the full list of items included as well as listing the results of the efficiency analyses and items retained in the short form

Constructs (IRM reliability)	Items		Short form
**AUTONOMY PROMOTION FOOD PARENTING DOMAIN**			
Child involvement 4 items (.87)	**In the past MONTH, how often did you… (answer for yourself only)** (Never, Rarely, Sometimes, Often, Always)		
	1	Have your child help prepare dinner meals	√
	2	Have your child help you prepare vegetable dishes	√
	3	Give your child a choice of vegetables to eat at dinner	√
	4	Ask your child’s opinion about what to make for meals	√
Autonomy support 14 items (.93)^a^	5	Serve healthy foods such as vegetables in a way your child likes to get your child to eat them	
	8	Help your child try a NEW vegetable or food by telling him or her that you like it and that he or she might like it also	√
	9	Say something nice to your child for tasting a NEW vegetable or food	
	10	Tell your child that colorful vegetables such as dark green, red, orange and purple vegetables are healthier than potatoes and corn	
	11	Read food labels with your child to help him or her choose healthier food or drinks	
	12	Explain that eating healthy food will give your child more energy	√
	13	Help your child eat or taste a vegetable by explaining how good it is for his or her health	√
	14	Tell your child that eating healthier food such as vegetables will help your child do better in school	
	16	Make your child think about whether he or she is full to teach your child to stop eating when full	
	17	Tell your child ideas on how he or she can make healthier food choices like eating more fruit or vegetables	√
	18	Tell your child reasons for the rules you make about food and the need to eat vegetables	√
	20	Tell your child that sweet or salty treats should only be eaten sometimes	
	21	Teach your child to eat food from all the food groups	
	22	Help your child set goals to eat more vegetables or other healthier food	
**CONTROL FOOD PARENTING DOMAIN**			
Restriction for weight 4 items (.79)	**To promote a healthy weight for your child, in the past month did you**… (Never, Rarely, Sometimes, Often, Always)		
	1	Keep your child away from specific sweet or salty treats (food or drinks)	√
	2	Keep a record of how much your child eats	√
	3	Not allow your child from taking second helpings at dinner	√
	5	Talk to your child about losing weight?	√
Coercive control 23 items (.96)^a^	**In the past MONTH, how often did you… (Answer for yourself only)** (Never, Rarely, Sometimes, Often, Always)		
	6	Give your child a sweet or salty treat to make your child feel better when your child is hurt	√
	7	Offer a sweet or salty treat when your child is worried or stressed to make your child feel better	√
	8	Offer a sweet or salty treat to calm your child down	√
	9	Give your child a sweet or salty treat to keep your child busy when you talking to another person or doing chores	√
	10	Give your child a sweet or salty treat to keep your child busy when he or she is bored, even if he or she is not hungry	
	11	Tell your child he or she will get dessert only if he or she tastes the vegetables you served	
	12	Promise your child dessert if he or she finishes their meal	
	13	Send your child to his or her room if they do not finish their meal	
	14	Reduce TV or videogame time if your child does not finish his or her meal	
	15	Reward your child with a sweet or salty treat for good behaviour	
	16	Take away dessert as punishment for bad behaviour	
	17	Offer your child a sweet or salty treat to make your child do something he or she does not want to do	√
	18	Take away TV or videogame time if your child does not eat the vegetables you served	
	19	Send your child to his or her room if your child refuses to eat the vegetables you served	
	20	Tell your child they will be punished if he or she eats a sweet or salty food or drink without asking you	
	25	Make your child stay at the table until all the food on his or her plate is eaten	
	26	Make your child eat more even if he or she says “I am full”	
	30	Make sure your child eats all his or her vegetables first at dinner time	
	32	You force your child to eat some vegetables every day	
	34	Hide vegetables in the food you serve as a way to get your child to eat more vegetables	
	35	Make your child feel bad about what he or she eats in order to get your child to eat healthier	
	36	Not allow your child to have sweet or salty treat at parties	
	37	Make your child eat a lighter meal, If your child ate more than usual at the earlier meal	
**STRUCTURE FOOD PARENTING DOMAIN**			
Nondirective Support 8 items (.88)^a^	**In the past MONTH, how often did you… (Answer for yourself only**) (Never, Rarely, Sometimes, Often, Always)		
	1	Eat or drink a healthy snack just because your child was around	
	3	Eat healthy portions while in front of your child (for example-take a smaller portion)	
	5	Show how much you enjoy eating vegetables while eating with your child	
	7	Encourage your child to eat the food as it is served, without picking the vegetables out	√
	8	Encourage your child to eat more at a meal if they don’t want to eat what is served but say they are not full	√
	9	Try to get your child to take a few more bites of their vegetables, without forcing them	√
	10	Encourage your child to eat more at dinner without pressuring him or her, if you feel your child has not eaten enough that day	√
	11	Try to get your child to taste a new vegetable (but not eat all of it) even if your child thinks he or she may not like it	√
Provide healthy eating opportunities 8 items (.87)^a^	12	Prepare your family’s meals mostly from scratch	
	14	Serve vegetables your child likes with meals	√
	15	Serve colourful vegetables (dark green, red, orange or purple vegetables) with meals	√
	22	Serve a vegetable multiple times even if your child has not liked it in the past	
	23	Serve your child at least 2 different vegetables (excluding potatoes or fries) at dinner meals	√
	24	Serve your child at least 5 different types of vegetables in a week?	√
	25	Serve at least 5 different fruit or berries (fresh or frozen) to your child in a week	√
	26	How much do you agree with this statement: I have consistently served a variety of vegetables to my child since he or she was 3 years old. (Strongly agree, Agree, Neutral, Disagree, Strongly disagree)	
Rules and limits 9 items (.88)^a^	28	You usually know how many sweet or salty treats your child eats or drinks at home	√
	29	You limit how often your child eats/drinks sweet or salty treats (i.e. chips, desserts, sugary drinks)	√
	31	You do not let your child drink soda or sugary drinks (e.g., sports drinks or fruit drinks)	
	33	You limit the portion size of sweet or salty treats your child eats	√
	34	You expect your child to eat the foods that you serve or not eat at all	
	35	If your child eats a sweet or salty treat, you expect the next snack to be healthy (e.g. to be a fruit)	√
	36	You expect your child to drink mostly water or milk with meals	
	37	You ask those who help take care of your child to limit the amount of sweet or salty treats they give to your child	
	39	You expect your child to ask for permission before he or she eats a sweet or salty treat or a sugary drink	√
Redirection 2 items (.67)	41	Encourage your child to only take a small portion, when your child asks for a less healthy treat	√
	44	Talk about food or drink options with your child and come to an agreement you are both happy with	√
Meal routines 4 items (.78)	47	Make your child eat dinner meals at the table	√
	50	Eat dinner together as a family (whole family)	√
	52	NOT allow your child to play, talk or text on the phone while eating dinner	√
	53	NOT allow your child to watch TV while eating dinner	√
Covert control 4 items (.81)	58	Keep sweet and salty treats out of your child’s reach	√
	59	Hide soda and sugary drinks in places where your child could not find them	√
	61	Throw away left over sweet or salty treats to discourage your child from eating them	√
	62	Not bring soda or sweet drinks into your home	√
Accommodating the child 5 items (.82)	63	Eat out at restaurants or get take-out food for meals with your child	√
	65	Give in and let your child have dessert, after you told him or her “no”	√
	67	Allow your child to skip meals (e.g., breakfast or lunch)	√
	69	Make only the foods your child asks for meals	√
	70	Buy your child a sweet or salty treat as a way to fill him or her up when you are on the go	√

IRM reliability = Empirical reliability computed from Item Response Modeling (IRM) which takes into account the ordinal nature of the data

^a^The IRM reliability for the short form is fixed at .80 for these constructs

## Discussion

In spite of multiple attempts to measure food parenting practices, this is the first study to use a rigorous psychometric process to refine and validate current conceptualizations of food parenting practices [6, 17]. The expert-informed food parenting practices conceptual framework [17], which guided the analyses, was supported by the CFA analyses. The CFA analyses for the three domains of parenting practices included the same 17 constructs as the conceptual framework with the exception of the access/availability and the permissive constructs which were renamed as measuring providing healthy eating opportunities and accommodating the child to better reflect the content of the final solution. The bi-factor item analyses and IRM analyses revealed that the conceptual framework could be simplified and three main domains of food parenting practices (autonomy promotion, control, and structure), could be operationalized with 11 unidimensional constructs (2–7 constructs per domain). The rigorous process used to assess the psychometric properties of the food parenting practices item bank highlighted the construct validity of the responses. This measurement study provides the needed tools for researchers interested in improving our understanding of how parenting practices influence child dietary behaviours.

The food parenting practices conceptual framework that guided the analyses [17] was informed by Vaughn and colleagues’ content map [6] which had a more complicated structure but hypothesized that the food parenting practices measured three higher-order constructs. While our analyses resulted in simplifying the expert-informed food parenting practices conceptual framework, many of the constructs could not be collapsed into the three main domains of parenting practices as hypothesized by Vaughn et al. [6]. Regrouping of the constructs into the main domains of parenting was achieved only for the control domain of food parenting practices, whereas only partial regrouping was possible for the other two domains of parenting practices. The analytical approach aimed to identify unidimensional constructs. That is, we aimed to simplify the conceptual framework only if the items assessed a single construct. While a higher level of simplification may have been achieved if we were looking to develop multidimensional constructs (constructs that collapsed distinct but related constructs into a single concept), this approach was not taken as multidimensional constructs are considered to be ambiguous and have been found to explain less variance and confound associations [31].

Some researchers may find the longer version (85 items) derived from the CFA results more relevant for their research as the level of detail may align better with their research questions. In fact, researchers who opt to use the long form will have the option of scoring the food parenting practices items using the CFA results or the results from the bifactor item analyses combined with the IRM analyses. However, for many researchers, the long form (85 items) will prove to be too lengthy and the short form (48 items) will likely provide an efficient alternative to measure the 11 food parenting practices constructs.

The conceptual framework on which the measurement work was based is comprehensive. However, the extent to which all of the 11 constructs are important to assess will depend on whether these constructs actually predict children’s dietary behaviours. Future refinement or simplification of the food parenting practices item bank may be achieved by gaining a greater understanding of whether all the food parenting practices constructs predict children’s dietary behaviours over time as well as determining whether all of the constructs are equally important to assess. Such work would help in assessing the construct validity of the food parenting practices item bank as well as help prioritize the most relevant constructs.

The development of the food parenting practices item bank followed the NIH PROMIS methodology to standardize the measurement of the constructs assessed by the item bank [18, 19]. Following the development of the item bank and the CFA analyses, the IRM analyses combined with computerized adaptive testing simulations helped develop a more efficient item bank (i.e., the short forms). Having shorter forms can facilitate use of the item bank by researchers. The next step in this line of research is to take advantage of having IRM calibrated item bank and use computerized adaptive testing methods to administer the item bank and provide more flexibility in item selection while maintaining the ability to compare scores across studies since scores would be scaled to a common metric.

This study has several strengths including following a rigorous process to operationalize the constructs and define their contents. In addition to using CFA method, this study supplemented these analyses with bi-factor item analyses and IRM methods to develop valid, efficient, and reliable ways of assessing food parenting practices. However, some limitations should be acknowledged. While a diverse group of parents were recruited from a web-based company, the calibration and invariance analyses were conducted in a sample of Canadian parents. The extent to which the psychometric properties are stable for different populations remains unknown. Future studies should also aim to replicate the psychometric analyses conduced in this study to test the stability of these analyses and test whether the constructs are stable across diverse populations. It was beyond the scope of this study to examine the criterion and predictive validity of the food parenting practices item bank. As such, future studies should examine the extent to which the constructs of the food parenting practice item bank measure what parents actually do and/or predict children’s dietary behaviours.

## Conclusions

Researchers examining food parenting practices have been challenged by inconsistencies in how different constructs are measured to describe the behaviours parents use to influence children’s dietary behaviours. The psychometric analyses presented here support the overall structure of the expert-endorsed food parenting conceptual framework while also providing a simplified structure [17]. The item bank provides efficient tools for researchers interested in examining how food parenting practices influence child dietary behaviours.

## Supplementary Information

**Additional file 1: Appendix A**: An expanded version of Table 3 that is shown in the manuscript. **Appendix B**: Food Parenting Item Bank Questionnaire. **Appendix C**.

## Data Availability

Please contact the first author (LCM at lmasse@bcchr.ubc.ca) for any questions about the study including data requests or study materials.

## Abbreviations

CFA: Confirmatory Factor Analysis
CFI: Comparative Fit Index
DIF: Differential Item Functioning
DRF: Differential Response Functioning
I-ECV: Explained Common Variance for a Single Item
IRM: Item Response Modeling
RMSEA: Steiger’s Root Mean Square Error of Approximation
WRMR: weighted root mean square residual
90% CI: 90% Confidence Intervals
