# Supplementary material for: Calibration of the food parenting practice (FPP) item bank: tools for improving the measurement of food parenting practices of parents of 5–12-year-old children
Source: Int J Behav Nutr Phys Act. 2020 Nov 16;17:140. doi: 10.1186/s12966-020-01049-9 (PMC7670656; doi:10.1186/s12966-020-01049-9)
Supplement: Supplementary file 1 — Additional file 1: Appendix A: An expanded version of Table 3 that is shown in the manuscript. Appendix B: Food Parenting Item Bank Questionnaire. Appendix C. [file 12966_2020_1049_MOESM1_ESM.docx]

**Appendix A: An expanded version of Table 3 that is shown in the manuscript.**

Full results from the Confirmatory Factor Analyses (CFA), Bi-factor item analyses, and Item Response Modeling analyses

| **Items** | | | | **Confirmatory Factor Analysis (CFA)** | | | | | | | | **Bi-Factor Item Analysis** | | | | | | | **Drop code** | |
| --- | --- | --- | --- | --- | --- | --- | --- | --- | --- | --- | --- | --- | --- | --- | --- | --- | --- | --- | --- | --- |
|  |  |  |  | **Constructs**  **(Cronbach α)** | | | | | **λ** | | | **Constructs** | | | **λ** | | **I-ECV** | |  |  |
| **AUTONOMY PROMOTION FOOD PARENTING DOMAIN** | | | | | | | | | | | | | | | | | | | | |
| 1 Have your child help prepare dinner meals | | | | Child  Involvement  (.75) | | | | | .87 | | | Child involvement | | | .22 | | .06 | |  | |
| 2 Have your child help you prepare vegetable dishes | | | |  |  |  |  |  | .93 | | |  |  |  | .27 | | .09 | |  | |
| 3 Give your child a choice of veggies to eat at dinner | | | |  |  |  |  |  | .59 | | |  |  |  | .27 | | .24 | |  | |
| 4 Ask your child’s opinion about what to make for meals | | | |  |  |  |  |  | .48 | | |  |  |  | .26 | | .37 | |  | |
| 5 Serve healthy foods (veggies) in the way child likes | | | | Encourage  (.77) | | | | | .65 | | | Autonomy support | | | .59 | | .83 | |  | |
| 6 Use games to make eating veggies or new food fun | | | |  |  |  |  |  | Drop | | |  |  |  |  | |  | | CFA | |
| 7 Other people they look up eat their veggies | | | |  |  |  |  |  | Drop | | |  |  |  |  | |  | | CFA | |
| 8 Tell child you like it and that he or she might like it also | | | |  |  |  |  |  | .83 | | |  |  |  | .74 | | .78 | |  | |
| 9 Say something nice if child taste a NEW vegetable / food | | | |  |  |  |  |  | .82 | | |  |  |  | .72 | | .78 | |  | |
| 10 Tell your child that colorful veggies are healthier | | | | Education  (.91) | | | | | .72 | | |  |  |  | .69 | | .91 | |  | |
| 11 Read food labels to help child choose healthier options | | | |  |  |  |  |  | .67 | | |  |  |  | .63 | | .81 | |  | |
| 12 Explain that eating healthy food gives more energy | | | |  |  |  |  |  | .80 | | |  |  |  | .85 | | .96 | |  | |
| 13 Explain how good it is to eat or taste vegetable | | | |  |  |  |  |  | .83 | | |  |  |  | .87 | | .97 | |  | |
| 14 Will do better in school by eating healthier/veggies | | | |  |  |  |  |  | .75 | | |  |  |  | .74 | | .98 | |  | |
| 15 Tell your child to eat less on days they are inactive | | | |  |  |  |  |  | Drop | | |  |  |  |  | |  | | CFA | |
| 16 Teach your child to stop eating when full | | | |  |  |  |  |  | .66 | | |  |  |  | .59 | | .65 | |  | |
| 17 Give child ideas on how to make healthy food choices | | | |  |  |  |  |  | .79 | | |  |  |  | .76 | | .88 | |  | |
| 18 Explain your reasons for food rules tell | | | |  |  |  |  |  | .76 | | |  |  |  | .73 | | .89 | |  | |
| 19 Play games that teach why healthy foods are good | | | |  |  |  |  |  | Drop | | |  |  |  |  | |  | | CFA | |
| 20 Explain that treats are sometimes food | | | |  |  |  |  |  | .63 | | |  |  |  | .62 | | .97 | |  | |
| 21 Teach your child to eat food from all the food groups | | | |  |  |  |  |  | .65 | | |  |  |  | .63 | | .91 | |  | |
| 22 Help child set goals to eat more veggies/healthier | | | |  |  |  |  |  | .76 | | |  |  |  | .70 | | .75 | |  | |
| **CFA correlations between constructs** | | | | | CIn | | Enc | | | Edu | |  | | | | | | | | |
| Child involvement (CIn) | | | | | 1 | |  | | |  | |  | | | | | | | | |
| Encourage (Enc) | | | | | .22 | | 1 | | |  | |  | | | | | | | | |
| Education (Edu) | | | | | .34 | | .88 | | | 1 | |  | | | | | | | | |
| **CONTROL FOOD PARENTING DOMAIN** | | | | | | | | | | | | | | | | | | | | |
| **Items** | | | | **Confirmatory Factor Analysis (CFA)** | | | | | | | | **Bi-Factor Item Analysis** | | | | | | | **Drop code** | |
|  |  |  |  | **Constructs**  **(Cronbach α)** | | | | | **λ** | | | **Constructs** | | | **λ** | | **I-ECV** | |  |  |
| 1 Keep away from specific sweet or salty treats | | | | Restrict for weight  (.80) | | | | | .52 | | | Restriction for weight | | | .34 | | .40 | |  | |
| 2 Keep a record of how much your child eats | | | |  |  |  |  |  | .86 | | |  |  |  | .62 | | .55 | |  | |
| 3 Not allow child to take second helpings at dinner | | | |  |  |  |  |  | .84 | | |  |  |  | .60 | | .52 | |  | |
| 4 Decide how much / how often your child eats | | | |  |  |  |  |  | .53 | | |  |  |  | .34 | | .26 | | LD | |
| 5 Talk to your child about dieting/loosing weight | | | |  |  |  |  |  | .88 | | |  |  |  | .63 | | .55 | |  | |
| 6 Give sweet or salty treat to make your child feel | | | | Use fiod to control negative emotions  (.95) | | | | | .90 | | | Coercive control | | | .81 | | .79 | |  | |
| 7 Offer a treat when child is worried/stressed | | | |  |  |  |  |  | .92 | | |  |  |  | .82 | | .77 | |  | |
| 8 Offer a treat to calm your child down | | | |  |  |  |  |  | .95 | | |  |  |  | .86 | | .84 | |  | |
| 9 Give treat when talking/doing chores | | | |  |  |  |  |  | .94 | | |  |  |  | .86 | | .86 | |  | |
| 10 Give treat to keep when child is bored | | | |  |  |  |  |  | .92 | | |  |  |  | .84 | | .82 | |  | |
| 11 Child gets dessert if tastes veggies served | | | | Threats & bribes  (.94) | | | | | .75 | | |  |  |  | .67 | | .69 | |  | |
| 12 Promise child dessert if finish meal | | | |  |  |  |  |  | .76 | | |  |  |  | .68 | | .70 | |  | |
| 13 Send child to room if does not finish meal | | | |  |  |  |  |  | .90 | | |  |  |  | .89 | | .98 | |  | |
| 14 Reduce TV/ videogame if child does not finish meal | | | |  |  |  |  |  | .80 | | |  |  |  | .74 | | .81 | |  | |
| 15 Reward good behaviours with a sweet or salty treat | | | |  |  |  |  |  | .76 | | |  |  |  | .71 | | .84 | |  | |
| 16 Take away dessert for bad behaviour | | | |  |  |  |  |  | .79 | | |  |  |  | .74 | | .84 | |  | |
| 17 Offer a treat to make child do something | | | |  |  |  |  |  | .88 | | |  |  |  | .85 | | .96 | |  | |
| 18 Take away TV/videogame if not eat veggies | | | |  |  |  |  |  | .90 | | |  |  |  | .86 | | .90 | |  | |
| 19 Send child to room for refusing to eat veggies | | | |  |  |  |  |  | .93 | | |  |  |  | .91 | | .99 | |  | |
| 20 Tell child will be punished for eating without asking | | | |  |  |  |  |  | .81 | | |  |  |  | .80 | | .99 | |  | |
| 21 Make child eat all the food on his or her plate | | | | Pressure to eat  (.79) | | | | | Drop | | |  |  |  |  | |  | | CFA | |
| 22 Make child finish all the veggies on plate | | | |  |  |  |  |  | Drop | | |  |  |  |  | |  | | CFA | |
| 23 Beg child to eat his or her veggies at dinner | | | |  |  |  |  |  | Drop | | |  |  |  |  | |  | | CFA | |
| 24 Make child try at least some veggies at dinner | | | |  |  |  |  |  | Drop | | |  |  |  |  | |  | | CFA | |
| 25 Make child stay at table until all the food is eaten | | | |  |  |  |  |  | .49 | | |  |  |  | .39 | | .96 | |  | |
| 26 Make child eat more even if s/he says “I am full” | | | |  |  |  |  |  | .72 | | |  |  |  | .51 | | .42* | |  | |
| 27 Physically struggle with child to eat meal | | | |  |  |  |  |  | .88 | | |  |  |  | .65 | | .54 | | I-ECV | |
| 28 Guilt child into eating his or her meal | | | |  |  |  |  |  | .84 | | |  |  |  | .62 | | .50 | | I-ECV | |
| 29 Show disappointment if child does not eat veggies | | | |  |  |  |  |  | .73 | | |  |  |  | .53 | | .48 | | I-ECV | |
| 30 Make sure child eats all veggies at dinner time | | | |  |  |  |  |  | .64 | | |  |  |  | .52 | | .99 | |  | |
| 31 You decide what foods your child eat for snacks | | | |  |  |  |  |  | Drop | | |  |  |  |  | |  | | CFA | |
| 32 You force child to eat some veggies every day | | | |  |  |  |  |  | .45 | | |  |  |  | .37 | | .97 | |  | |
| 33 Decide when child is allowed to eat a snack | | | | Intrusive control  (.79) | | | | | Drop | | |  |  |  |  | |  | | CFA | |
| 34 Hide veggies in the food you serve | | | |  |  |  |  |  | .68 | | |  |  |  | .64 | | .96 | |  | |
| 35 Make child feel bad about what s/he eats | | | |  |  |  |  |  | .84 | | |  |  |  | .78 | | .92 | |  | |
| 36 Not allow child to have a treat at parties | | | |  |  |  |  |  | .78 | | |  |  |  | .72 | | .82 | |  | |
| 37 Make a lighter meal, if child ate more earlier | | | |  |  |  |  |  | .66 | | |  |  |  | .61 | | .74 | |  | |
| **CFA correlations between constructs** | Rwe | Nem | | | T&B | | Pre | | | Ico | |  | | | | | | | | |
| Restrict for weight control (Rwe) | 1 |  | | |  | |  | | |  | |  | | | | | | | | |
| Negative emotion (Nem) | .55 | 1 | | |  | |  | | |  | |  | | | | | | | | |
| Treats &bribes (T&B) | .58 | .89 | | | 1 | |  | | |  | |  | | | | | | | | |
| Pressure (Pre) | .76 | .58 | | | .67 | | 1 | | |  | |  | | | | | | | | |
| Intrusive control (Ico | .72 | .84 | | | .88 | | .70 | | | 1 | |  | | | | | | | | |
| **STRUCTURE FOOD PARENTING DOMAIN** | | | | | | | | | | | | | | | | | | | | |
| **Items** | | | | **Confirmatory Factor Analysis (CFA)** | | | | | | | | **Bi-Factor Item Analysis** | | | | | | | **Drop code** | |
|  |  |  |  | **Constructs**  **(Cronbach α)** | | | | | **λ** | | | **Constructs** | | | **λ** | | **I-ECV** | |  | |
| 1 Eat healthy snacks when child is around | | | | Modeling  (.69) | | | | | .66 | | | Non-directive support | | | .57 | | .87 | |  | |
| 2 Buy a healthy meal when with child | | | |  |  |  |  |  | Drop | | |  |  |  |  | |  | | CFA | |
| 3 Eat healthy portions while with child | | | |  |  |  |  |  | .68 | | |  |  |  | .47 | | .18* | |  | |
| 4 Eat veggies when child is with you | | | |  |  |  |  |  | Drop | | |  |  |  |  | |  | | CFA | |
| 5 Enjoy eating veggies when with child | | | |  |  |  |  |  | .76 | | |  |  |  | .64 | | .86 | |  | |
| 6 Gently remind child to eat veggies | | | | Prompt to eat  (.83) | | | | | Drop | | |  |  |  |  | |  | | CFA | |
| 7 Encourage child to eat food as served | | | |  |  |  |  |  | .68 | | |  |  |  | .70 | | 1.00 | |  | |
| 8 Encourage child to eat more if not full | | | |  |  |  |  |  | .73 | | |  |  |  | .68 | | .80 | |  | |
| 9 Get child to eat more veggies | | | |  |  |  |  |  | .79 | | |  |  |  | .77 | | .97 | |  | |
| 10 Encourage child to eat more | | | |  |  |  |  |  | .76 | | |  |  |  | .72 | | .75 | |  | |
| 11 Get child to taste a new veggies | | | |  |  |  |  |  | .74 | | |  |  |  | .78 | | .95 | |  | |
| 12 Family meals prepared from scratch | | | | Meal preparation (.68) | | | | | .59 | | | Providing healthy eating opportunities | | | .59 | | .94 | |  | |
| 13 Serve at least two veggies for dinner | | | |  |  |  |  |  | Drop | | |  |  |  |  | |  | | CFA | |
| 14 Serve veggies your child likes | | | |  |  |  |  |  | .72 | | |  |  |  | .68 | | .95 | |  | |
| 15 Serve colourful veggies with meals | | | |  |  |  |  |  | .77 | | |  |  |  | .81 | | .90 | |  | |
| 16 Serve pre-packaged dinner | | | |  |  |  |  |  | Drop | | |  |  |  |  | |  | | CFA | |
| 17 Serve dessert sweet treat) after dinner | | | |  |  |  |  |  | Drop | | |  |  |  |  | |  | | CFA | |
| 18 Provide only water/milk at meal/snack | | | |  |  |  |  |  | Drop | | |  |  |  |  | |  | | CFA | |
| 19 Serve/make food that includes less fat | | | |  |  |  |  |  | Drop | | |  |  |  |  | |  | | CFA | |
| 20 Serve/make food that include less sugar | | | |  |  |  |  |  | Drop | | |  |  |  |  | |  | | CFA | |
| 21 Child to eat food by serving it again | | | | Exposure to variety/ selection  (.75) | | | | | Drop | | |  |  |  |  | |  | | CFA | |
| 22 Serve a vegetable multiple times | | | |  |  |  |  |  | .67 | | |  |  |  | .59 | | .96 | |  | |
| 23 Serve child at least 2 different veggies | | | |  |  |  |  |  | .68 | | |  |  |  | .71 | | .99 | |  | |
| 24 Serve child at least 5 different veggies | | | |  |  |  |  |  | .77 | | |  |  |  | .77 | | .75 | |  | |
| 25 Serve at least 5 different fruits | | | |  |  |  |  |  | .70 | | |  |  |  | .64 | | .77 | |  | |
| 26 Consistently served veggies since young | | | |  |  |  |  |  | .45 | | |  |  |  | .47 | | 1.00 | |  | |
| 27 Foods from different countries / cultures | | | |  |  |  |  |  | .47 | | |  |  |  | .42 | | .89 | | DRF | |
| 28 Know how many treats child has | | | | Rules and limits (.86) | | | | | .69 | | | Rules and limits | | | NA | | NA | |  | |
| 29 Limit sweet or salty treats | | | |  |  |  |  |  | .81 | | |  |  |  | NA | | NA | |  | |
| 30 Only let child snack on healthy food | | | |  |  |  |  |  | Drop | | |  |  |  | NA | | NA | | CFA | |
| 31 Do not let child drink soda/sugary drinks | | | |  |  |  |  |  | .61 | | |  |  |  | NA | | NA | |  | |
| 32 Keep track amount of veggies child eats | | | |  |  |  |  |  | Drop | | |  |  |  | NA | | NA | | CFA | |
| 33 Limit size of treats child eats | | | |  |  |  |  |  | .82 | | |  |  |  | NA | | NA | |  | |
| 34 Expect child to eat what you serve | | | |  |  |  |  |  | .40 | | |  |  |  | NA | | NA | |  | |
| 35 If child eats a treat, expect next healthy | | | |  |  |  |  |  | .72 | | |  |  |  | NA | | NA | |  | |
| 36 Expect child to drink mostly water/milk | | | |  |  |  |  |  | .68 | | |  |  |  | NA | | NA | |  | |
| 37 Ask those who care for child to limit treats | | | |  |  |  |  |  | .64 | | |  |  |  | NA | | NA | |  | |
| 38 Not allow to eat/ drink certain times | | | |  |  |  |  |  | Drop | | |  |  |  | NA | | NA | | CFA | |
| 39 Child ask permission before eating treat | | | |  |  |  |  |  | .73 | | |  |  |  | NA | | NA | |  | |
| 40 Suggest child eat fruit/veggies not a treat | | | | Redirection (.61) | | | | | Drop | | | Redirection | | | NA | | NA | | CFA | |
| 41 Encourage to take small portion for treats | | | |  |  |  |  |  | .72 | | |  |  |  | NA | | NA | |  | |
| 42 Encourage to split treats with someone | | | |  |  |  |  |  | Drop | | |  |  |  | NA | | NA | | CFA | |
| 43 Give something else to do if ask for treat | | | |  |  |  |  |  | Drop | | |  |  |  | NA | | NA | | CFA | |
| 44 Talk/agree about treat options with child | | | |  |  |  |  |  | .66 | | |  |  |  | NA | | NA | |  | |
| 45 Agree on how much veggies child eats | | | |  |  |  |  |  | Drop | | |  |  |  | NA | | NA | | CFA | |
| 46 Agree about treats child is allowed | | | |  |  |  |  |  | Drop | | |  |  |  | NA | | NA | | CFA | |
| 47 Make child eat dinner meals at the table | | | |  |  |  |  |  | .81 | | |  |  |  | NA | | NA | |  | |
| 48 Let child know when dinner will be ready | | | |  |  |  |  |  | Drop | | |  |  |  | NA | | NA | | CFA | |
| 49 Eat dinner with child | | | | Meal routines  (.74) | | | | | Drop | | | Meal routines | | | NA | | NA | | CFA | |
| 50 Eat dinner together as a family | | | |  |  |  |  |  | .73 | | |  |  |  | NA | | NA | |  | |
| 51 Child eat dinner meal before the adults | | | |  |  |  |  |  | Drop | | |  |  |  | NA | | NA | | CFA | |
| 52 No play/talk/text while eating | | | |  |  |  |  |  | .70 | | |  |  |  | NA | | NA | |  | |
| 53 No TV while eating | | | |  |  |  |  |  | .66 | | |  |  |  | NA | | NA | |  | |
| 54 Keep fruit where child can reach | | | | Covert control (.76) | | | | | Drop | | | Covert control | | | NA | | NA | | CFA | |
| 55 Have ready-to-eat veggies child can grab | | | |  |  |  |  |  | Drop | | |  |  |  | NA | | NA | | CFA | |
| 56 Have several fruit at home for your child | | | |  |  |  |  |  | Drop | | |  |  |  | NA | | NA | | CFA | |
| 57 Buy sweet or salty treats when shopping | | | |  |  |  |  |  | Drop | | |  |  |  | NA | | NA | | CFA | |
| 58 Keep treats out of your child’s reach | | | |  |  |  |  |  | .80 | | |  |  |  | NA | | NA | |  | |
| 59 Hide sugary drinks so child cannot find | | | |  |  |  |  |  | .81 | | |  |  |  | NA | | NA | |  | |
| 60 Not bring treats into your home | | | |  |  |  |  |  | Drop | | |  |  |  | NA | | NA | | CFA | |
| 61 Throw away left over treats | | | |  |  |  |  |  | .68 | | |  |  |  | NA | | NA | |  | |
| 62 Not bring sugary drinks into home | | | |  |  |  |  |  | .59 | | |  |  |  | NA | | NA | | LD* | |
| 63 Eat out or get take-out food for meals | | | | Accommodating the child (.81) | | | | | .56 | | | Accommodating the child | | | NA | | NA | |  | |
| 64 Take something if child doesn’t like meal | | | |  |  |  |  |  | Drop | | |  |  |  | NA | | NA | | CFA | |
| 65 Give in and let child have dessert | | | |  |  |  |  |  | .74 | | |  |  |  | NA | | NA | |  | |
| 66 Offer seconds without being asked | | | |  |  |  |  |  | Drop | | |  |  |  | NA | | NA | | CFA | |
| 67 Allow child to skip meals | | | |  |  |  |  |  | .76 | | |  |  |  | NA | | NA | |  | |
| 68 Let child have a treat when s/he wants | | | |  |  |  |  |  | Drop | | |  |  |  | NA | | NA | | CFA | |
| 69 Make only the foods child asks for meals | | | |  |  |  |  |  | .73 | | |  |  |  | NA | | NA | |  | |
| 70 Buy a treat to fill child when on the go | | | |  |  |  |  |  | .76 | | |  |  |  | NA | | NA | |  | |
| **CFA correlations between constructs** | | | Mod | | | Pro | | Mpr | | | Exp | | R&L | Red | | Rou | | Cco | | Ach |
| Model (Mod) | | | 1. | | |  | |  | | |  | |  |  | |  | |  | |  |
| Prompt to eat (Pro) | | | .79 | | | 1 | |  | | |  | |  |  | |  | |  | |  |
| Meal preparation (Mpr) | | | .31 | | | .19 | | 1 | | |  | |  |  | |  | |  | |  |
| Exposure (Exp) | | | .31 | | | .20 | | .97 | | | 1 | |  |  | |  | |  | |  |
| Rules & Limits (R&L) | | | .33 | | | .25 | | ,56 | | | .55 | | 1 |  | |  | |  | |  |
| Redirection (Red) | | | .43 | | | .34 | | .36 | | | .43 | | .63 | 1 | |  | |  | |  |
| Meal routines (Rou) | | | .23 | | | .15 | | .65 | | | .55 | | .63 | .28 | | 1 | |  | |  |
| Convert control (Cco) | | | .58 | | | .58 | | .15 | | | .18 | | .38 | .45 | | .01 | | 1 | |  |
| Accommodate child (Ach) | | | .20 | | | .28 | | -.15 | | | -.05 | | -.17 | .48 | | -.35 | | .40 | | 1 |

Drop code: DRF: Deleted since item is not invariant (significant Differential Response Functioning); I-ECV (explained common variance for a single item) is less than .50 I-ECV; and LD = LD Local dependence.

* Kept from a content perspective even though the I-ECV is low or LD is present.

**Appendix B: Food Parenting Item Bank Questionnaire**

We want to know what you are **currently doing** for your **[X year old child]**. The questions ask if **YOU** are doing these things now, **NOT** what you might do in the future and **NOT** what others who help care for your child are doing including your spouse/common-law partner.

**Sweet and salty treats** refer to treats, snacks and desserts. These may include potato chips, corn chips, popcorn, cakes, cookies, donuts, pies, ice cream, chocolate and candies.

| **AUTONOMY PROMOTION ITEMS**  * items included in the short form |
| --- |

In the past **MONTH**, how often did you… (Answer for yourself only).

**[Child involvement 1*]** Have your child help prepare dinner meals

**[Child involvement 2*]** Have your child help you prepare vegetable dishes

**[Child involvement 3*]** Give your child a choice of vegetables to eat at dinner

**[Child involvement 4*]** Ask your child’s opinion about what to make for meals

**[Autonomy support 5]** Serve healthy foods such as vegetables in a way your child likes to get your child to eat them

**[Autonomy support 8*]** Help your child want to try a NEW vegetable or food by telling him or her that you like it and that he or she might like it also

**[Autonomy support 9]** Say something nice to your child for tasting a NEW vegetable or food

**[Autonomy support 10]** Tell your child that colorful vegetables such as dark green, red, orange and purple vegetables are healthier than potatoes and corn

**[Autonomy support 11]** Read food labels with your child to help him or her choose healthier food or drinks

**[Autonomy support 12*]** Explain that eating healthy food will give your child more energy

**[Autonomy support 13*]** Help your child eat or taste a vegetable by explaining how good it is for his or her health

**[Autonomy support 14]** Tell your child that eating healthier food such as vegetables will help your child do better in school

**[Autonomy support 16]** Make your child think about whether he or she is full to teach your child to stop eating when full

**[Autonomy support 17*]** Tell your child ideas on how he or she can make healthier food choices like eating more fruit or vegetables

**[Autonomy support 18*]** Tell your child reasons for the rules you make about food and the need to eat vegetables

**[Autonomy support 20]** Tell your child that sweet or salty treats should only be eaten sometimes

**[Autonomy support 21]** Teach your child to eat food from all the food groups

**[Autonomy support 22]** Help your child set goals to eat more vegetables or other healthier food

**[RESPONSE FORMAT]**

Never

Rarely

Sometimes

Often

Always

| **CONTROL ITEMS**  * items included in the short form |
| --- |

The next questions ask about strategies you may use to promote healthy weight.

To promote a healthy weight for your child, in the past month did you…

**[Restrict for weight control 1*]** Keep your child away from specific sweet or salty treats (food or drinks)

**[Restrict for weight control 2*]** Keep a record of how much your child eats

**[Restrict for weight control 3*]** Not allow your child from taking second helpings at dinner

**[Restrict for weight control 5*]** Talk to your child about losing weight

**[RESPONSE FORMAT]**

Never

Rarely

Sometimes

Often

Always

In the past **MONTH**, how often did you… (Answer for yourself only).

**[Coercive control 6*]** Give your child a sweet or salty treat to make your child feel better when your child is hurt

**[Coercive control 7*]** Offer a sweet or salty treat when your child is worried or stressed to make your child feel better

**[Coercive control 8*]** Offer a sweet or salty treat to calm your child down

**[Coercive control 9*]** Give your child a sweet or salty treat to keep your child busy when you talking to another person or doing chores

**[Coercive control 10]** Give your child a sweet or salty treat to keep your child busy when he or she is bored, even if he or she is not hungry

**[Coercive control 11]** Tell your child he or she will get dessert only if he or she tastes the vegetables you served

**[Coercive control 12]** Promise your child dessert if he or she finishes their meal

**[Coercive control 13]** Send your child to his or her room if they do not finish their meal

**[Coercive control 14]** Reduce TV or videogame time if your child does not finish his or her meal

**[Coercive control 15]** Reward your child with a sweet or salty treat for good behaviour

**[Coercive control 16]** Take away dessert as punishment for bad behaviour

**[Coercive control 17*]** Offer your child a sweet or salty treat to make your child do something he or she does not want to do

**[Coercive control 18]** Take away TV or videogame time if your child does not eat the vegetables you served

**[Coercive control 19]** Send your child to his or her room if your child refuses to eat the vegetables you served

**[Coercive control 20]** Tell your child they will be punished if he or she eats a sweet or salty food or drink without asking you

**[Coercive control 25*]** Make your child stay at the table until all the food on his or her plate is eaten

**[Coercive control 26]** Make your child eat more even if he or she says “I am full”

**[Coercive control 30]** Make sure your child eats all his or her vegetables first at dinner time

**[Coercive control 32]** You force your child to eat some vegetables every day

**[Coercive control 34]** Hide vegetables in the food you serve as a way to get your child to eat more vegetables

**[Coercive control 35]** Make your child feel bad about what he or she eats in order to get your child to eat healthier

**[Coercive control 36]** Not allow your child to have sweet or salty treat at parties

**[Coercive control 37]** Make your child eat a lighter meal, If your child ate more than usual at the earlier meal

**[RESPONSE FORMAT]**

Never

Rarely

Sometimes

Often

Always

| **STRUCTURE ITEMS**  * items included in the short form |
| --- |

In the past **MONTH**, how often did you… (Answer for yourself only).

**[Nondirective support 1]** Eat or drink a healthy snack just because your child was around

**[Nondirective support 3]** Eat healthy portions while in front of your child (for example-take a smaller portion)

**[Nondirective support 5]** Show how much you enjoy eating vegetables while eating with your child

**[Nondirective support 7*]** Encourage your child to eat the food as it is served, without picking the vegetables out

**[Nondirective support 8*]** Encourage your child to eat more at a meal if they don't want to eat what is served but say they are not full

**[Nondirective support 9*]** Try to get your child to take a few more bites of their vegetables, without forcing them

**[Nondirective support 10*]** Encourage your child to eat more at dinner without pressuring him or her, if you feel your child has not eaten enough that day

**[Nondirective support 11*]** Try to get your child to taste a new vegetable (but not eat all of it) even if your child thinks he or she may not like it

**[Provide healthy eating opportunities 12]** Prepare your family’s meals **mostly** from scratch

**[Provide healthy eating opportunities 14*]** Serve vegetables your child likes with meals

**[Provide healthy eating opportunities 15*]** Serve colourful vegetables (dark green, red, orange or purple vegetables) with meals

**[Provide healthy eating opportunities 22]** Serve a vegetable multiple times even if your child has not liked it in the past

**[Provide healthy eating opportunities 23*]** Serve your child at least 2 different vegetables (excluding potatoes or fries) at dinner meals

**[Provide healthy eating opportunities 24*]** Serve your child at least 5 different types of vegetables in a week

**[Provide healthy eating opportunities 25*]** Serve at least 5 different fruit or berries (fresh or frozen) to your child in a week

**[RESPONSE FORMAT]**

Never

Rarely

Sometimes

Often

Always

**[Provide healthy eating opportunities 26]** How much do you agree with this statement: I have consistently served a variety of vegetables to my child since he or she was 3 years old. *Choose one.*

Strongly agree

Agree

Neutral

Disagree

Strongly disagree

In the past **MONTH**, how often did you… (Answer for yourself only).

**[Rules and limits 28*]** You usually know how many sweet or salty treats your child eats or drinks at home

**[Rules and limits 29*]** You limit how often your child eats/drinks sweet or salty treats (i.e. chips, desserts, sugary drinks)

**[Rules and limits 31]** You do not let your child drink soda or sugary drinks (e.g., sports drinks or fruit drinks)

**[Rules and limits 33*]** You limit the portion size of sweet or salty treats your child eats

**[Rules and limits 34]** You expect your child to eat the foods that you serve or not eat at all

**[Rules and limits 35*]** If your child eats a sweet or salty treat, you expect the next snack to be healthy (e.g. to be a fruit)

**[Rules and limits 36]** You expect your child to drink mostly water or milk with meals

**[Rules and limits 37]** You ask those who help take care of your child to limit the amount of sweet or salty treats they give to your child

**[Rules and limits 39*]** You expect your child to ask for permission before he or she eats a sweet or salty treat or a sugary drink

**[Redirection 41*]** Encourage your child to only take a small portion, when your child asks for a less healthy treat

**[Redirection 44*]** Talk about food or drink options with your child and come to an agreement you are both happy with

**[Meal routines 47*]** Make your child eat dinner meals at the table

**[Meal routines 50*]** Eat dinner together as a family (whole family)

**[Meal routines 52*]** NOT allow your child to play, talk or text on the phone while eating dinner

**[Meal routines 53*]** NOT allow your child to watch TV while eating dinner

**[Covert control 58*]** Keep sweet and salty treats out of your child’s reach

**[Covert control 59*]** Hide soda and sugary drinks in places where your child could not find them

**[Covert control 61*]** Throw away left over sweet or salty treats to discourage your child from eating them

**[Covert control 62*]** Not bring soda or sweet drinks into your home

**[Accomodating the child 63*]** Eat out at restaurants or get take-out food for meals with your child

**[Accomodating the child 65*]** Give in and let your child have dessert, after you told him or her “no”

**[Accomodating the child 67*]** Allow your child to skip meals (e.g., breakfast or lunch)

**[[Accomodating the child 69*]** Make only the foods your child asks for meals

**[Accommodating the child 70*]** Buy your child a sweet or salty treat as a way to fill him or her up when you are on the go

**[RESPONSE FORMAT]**

Never

Rarely

Sometimes

Often

Always
